# SARS-CoV-2 Infection and Candidate Biomarkers

**DOI:** 10.5152/eurasianjmed.2022.22305

**Published:** 2022-12-01

**Authors:** Sevgi Karabulut Uzunçakmak

**Affiliations:** Health Services Vocational School, Bayburt University, Bayburt, Turkey

**Keywords:** SARS-CoV-2, COVID-19, biomarkers, infectious disease

## Abstract

Severe acute respiratory syndrome coronavirus 2 is a virus that can still infect individuals and whose deadly effects continue despite the current vaccines and drugs. Since 2019, many studies on the pathogenesis of the disease have been completed and continue to be done. In addition to the diagnosis and treatment of the disease, many molecules that can be markers of the disease have been investigated. In the early stages of the pandemic, many nonspecific and infection-related laboratory findings and chest computed tomography were used to obtain information about the diagnosis of the disease. The more individual molecules became associated with the disease yet. The purpose of this review is to summarize the impact and role of many molecules associated with coronavirus disease-2019 infection that have been previously used and newly revealed. Numerous studies are summarized in this review. The obtained data show that previously used laboratory findings and new potential biomarkers are not specific to the disease. New potential biomarkers have been associated with the severity of the disease itself, as can be seen with lung imaging and even with routine laboratory findings. One of the important points that are seen frequently in studies is that the effectiveness of these molecules has been shown not only in coronavirus disease-2019 infection but also in many other diseases. This removes the pathogenesis of the disease from being a unique mechanism created by the Severe acute respiratory syndrome coronavirus 2 and provides a general perspective formed by viral or bacterial infections. However, there are still many molecular changes that need to be investigated. Future studies will continue to update themselves with the mutations of the virus.

**Main Points**

- A major molecular mechanism specific to COVID-19 infection has yet to be elucidated.
- The findings obtained are very valuable as a part of this main mechanism.
- Data from studies show that biomarkers are not specific for a single disease or mechanism.
- When evaluating these biomarkers, scientists and healthcare professionals should keep in mind that more than 1 molecular mechanism time may have commonalities and should consider many possibilities.

## Introduction

Severe acute respiratory syndrome virus 2 (SARS-CoV-2), which caused the coronavirus disease-2019 (COVID-19) infection that spread from China to the world in 2019, is still a virus that shows its effect all over the world and can pose a threat to public health. The virus spreads easily from infective individuals to others through the respiratory tract. Infected people may show symptoms such as fever, myalgia, fatigue, cough, shortness of breath, headache, and loss of smell and taste.^1^ The severity of the disease varies from patient to patient, depending on many additional factors.^1^ Coronavirus disease-2019 disease may present with clinically mild symptoms such as a mild upper respiratory tract disease or an asymptomatic infection in some patients.^1^ Some patients may have lung damage, need intensive care, and even go into a process leading to death.^1^

Virus particles spreading from respiratory mucosal cells to other cells activate the immune system and lead to cytokine storms.^2^ Lymphocytopenia, is part of the this cytokine storm, was frequently observed in patients with COVID-19 infection, especially in patients with severe disease^2,3^ .^4^ Cytokine storm causes the release of proinflammatory cytokines such as interleukin-6 (IL-6), tumor necrosis factor-α (TNF-α), IL-2, and nitric oxide.^4,5^ The increased amount of cytokine impairs vascular permeability. This brings along many pathological processes such as disruption of tissue perfusion, endothelial damage, and thrombus formation.^6,7^ Increased vascular permeability results in acute respiratory failure due to the accumulation of liquid in the lung tissue.^8^ Exacerbated cytokines in the lungs cause alveolar septal fibrous proliferation, alveolar epithelial damage, and hyaline membrane formation and as a result hypoxic respiratory failure.^8,9^ Coronavirus disease-2019 patients often develop macrophage activation syndrome (MAS), which exacerbates the disease.^10,11^ MAS caused by severe cytokine storm, culminating from a complex interplay of genetics, immunodeficiency, infectious triggers, and dominant innate immune effector responses. Macrophage activation syndrome is not specific to COVID-19 infection; however, they have common features such as macrophage overactivation and cytokine storm.^12^ Macrophage activation syndrome and cytokine storm are a result of increased cytokine amount and leukocyte infiltration caused by tissue damage from SARS-CoV-2.^13^ Macrophage activation syndrome causes inflammation, tissue damage, organ failure, and high mortality rate.^12^

Some of the laboratory findings frequently used at the beginning of the pandemic are summarized below. Although these parameters are not specific to the disease, they have been used to obtain information about disease progression and severity. Significant associations have also been observed between lymphopenia and poor patient prognosis.^12^ A higher incidence of lymphopenia has been observed in COVID-19 patients with severe disease compared to moderately ill COVID-19 patients.^12^ Decreased CD8+ T cell, B cell, CD4+ T cell, and naturel killer cell counts and basophil, eosinophil, and monocytes were also observed in COVID-19 patients.^14^ Neutrophilia is another immune cell alteration observed in severe COVID-19 patients.^12^

C-reactive protein (CRP), procalcitonin (PCT), fibrinogen, and ferritin are positive acute-phase reactants because their expression is increased during inflammation.^15^ Since the amount of proteins such as albumin and transferrin decreases during inflammation, these are called negative acute-phase reactants.^15^ In systemic inflammation, CRP is secreted from the liver, and its level rises rapidly. C-reactive protein is used to follow many disease progressions as well as COVID-19 infections.^16,17^ Increased CRP value was associated with disease and disease severity. Comorbid diseases also contribute to high CRP levels.^18^ C-reactive protein values showed correlation with fibrinogen, ferritin, and pulmonary function tests.^19^ Coagulation derangement is a factor that is seen together with endothelial damage caused by the SARS-CoV-2 virus and has an important role in the course of the disease. d-dimer is a product of fibrinolysis, and its amount increases as a result of the degradation of the clot. Due to the coagulation dysregulation that develops in COVID-19 infection, blood d-dimer levels are elevated and are generally associated with poor patient prognosis.^5,19^ Plasma fibrinogen level was also used as another biomarker to predict blood coagulation dysregulation and disease severity of COVID-19 infection.^12^ Procalcitonin is a positive acute-phase reactant, and its level in serum may change in viral or bacterial infections.^20^ The PCT level was also used as a marker for COVID-19 disease, and the decreased PCT level was evaluated as a result of treatment efficacy.^20^ Vitamin D exerts an anti-inflammatory effect by suppressing T helper 1, which is an important cell in the cytokine storm.^21^ It has also been reported that angiotensin-converting enzyme 2 (ACE2) receptors increase with vitamin D, thus facilitating SARS-CoV-2 infection.^22^ Coronavirus disease-2019 patients with acute respiratory distress syndrome (ARDS) showed lower levels of vitamin D than control groups.^2^ Vitamin D may be considered more of an inflammation suppressor. Platelet count as laboratory finding was associated with disease severity for patients in the intensive care unit.^23,24^ Decreased platelet count was reported in COVID-19 patients^1^ and related to mortality.^25^ Microthrombus formation in the lung vessels may be a result of endothelial dysfunction and lung damage.^26^ Numerous laboratory findings such as these were biomarkers for the course of the disease albeit nonspecific for COVID-19 infection.

The drugs used in the treatment of COVID-19 infection or the disease itself have also caused significant changes in liver enzyme levels. Liver enzymes including transaminases gamma-glutamyl transferase were found to be increased in COVID-19 patients who develop MAS compared to COVID-19 patients who do not develop MAS.^11^ Higher alkaline phosphatase levels were detected in severe COVID-19 patients compared to moderately severe patients.^12^ One of the organs affected by the infection is the kidneys, and the biomarkers of the kidneys alone are informative about the prognosis of the patient. Serum creatinine level was found to be relatively high in COVID-19 patients who developed MAS compared to those who did not.^11^

Molecular testing of respiratory fluids obtained from patients, showing the presence of the virus, was used to prove whether the patients were infected with the virus even if they did not show any symptoms. The test used to diagnose the disease was used all over the world as a standard, but the prognosis and treatment of the disease at the post-diagnosis stage was carried out on nonspecific markers.^3^ Laboratory findings, lung imaging, and other clinical findings that are nonspecific to the disease have been used for the course of the disease.^3^ In this review, studies involving numerous potential biomarkers associated with COVID-19 disease and disease course are summarized.

### Candidate Biomarkers

Laboratory tests are used to obtain information about the health status, disease, and/or prognosis of diseases. Some values are used only for risk assessment, while others provide precise information about the diagnosis and progression of the disease. Especially in the early stages of the pandemic for COVID-19 infection, only nonspecific laboratory tests, lung imaging, and clinical findings were used as informative factors for the course of the disease.^1^ Among the frequently used laboratory findings are white blood cell count, neutrophil, lymphocyte, platelet, ferritin, d-dimer, CRP, and troponin values.^19^ As a result of clinical and laboratory studies on the disease, we have seen that many factors such as molecular mechanism changes, tissue damage, immunopathological changes, alteration in lipoprotein metabolism, and viral load contribute to the course of the disease.^27^

Although the parameters used routinely were not specific to the disease, they were informative about the course of the disease. However, as all diseases have their own molecular changes, the specific mechanisms underlying the COVID-19 infection need to be clarified. Moreover, more practical and easily applicable tests and biomarkers are needed for the diagnosis and course of the disease. Numerous studies conducted for this purpose have revealed that many molecules that may be specific to the disease change with it.^28^ Numerous potential biomarkers in different categories that may be associated with COVID-19 infection from different studies and their functions are summarized below.

### Genetic Predisposition

Although the severity of COVID-19 disease is a result of many pathophysiological processes, the underlying genetic predisposition has an important role in the course of the disease. Some genetic differences may lead patients to develop the disease and have the disease more severely. SARS-CoV-2 virus uses the ACE2 receptor for infection and transmembrane serine protease TMPRSS2 for SARS-CoV-2 spike (S) protein priming.^29^ TMPRSS2 rs12329760 and ACE2 rs2285666 single-nucleotide polymorphisms were related to COVID-19 disease severity.^30^ In another study also, ACE2 rs2285666 polymorphism was investigated. They found that the GG genotype or G-allele was associated with a 2-fold increased SARS-CoV-2 infection risk and a 3-fold increased risk severity of disease or COVID-19-caused death.^31^ Ponti et al^32^ suggested that MTHFR C677 T polymorphism may impact the severity of COVID-19 infection. Interferon (IFN) lambda 4 (IFNL4) is an essential antiviral protein with activity against RNA viruses.^33^ In the study by Saponi-Cortes et al.^34^ the IFNL4 rs12979860 T allele was found to be relatively higher in COVID-19 patients than in other individuals without the disease. Interleukin-6 is the cytokine produced by macrophages and plays an essential role in the formation of MAS and has been associated with disease severity in COVID-19 patients.^10^ Interlleukin-6 can alter vascular permeability, causing microthrombus formation, endothelial damage, and impaired tissue perfusion.^35^ In their study, Kerget et al^36^ showed that COVID-19 patients with the IL-6 (–174 G/C) genotype have lower serum IL-6 levels than patients with the IL-6 (–174 GG) genotype. The prevalence of MAS was also found to be higher in COVID-19 patients with the IL-6 (–174 GG) genotype with high IL-6 levels. Kerget et al^10^ also showed the existence of a significant relationship between increased IL-6 level and MAS and ARDS. Genetic disposition may be evaluated not only for the genes summarized above but perhaps for all genes that play a role in the immune system. Thus, individuals with a genetically high risk of disease can be identified and precautions can be taken.

### Endothelial Damage

Endothelial dysfunction in COVID-19 infection is seen in most patients.^37^ In COVID-19 patients, autopsy endothelial cell damage and apoptosis were observed in blood vessels.^38,39^ Endothelial dysfunction biomarkers were found to be relatively higher in COVID-19 patients than in healthy controls and were associated with severe disease.^40,41^ Nuclear factor erythroid 2–related factor 2 (NRF2) is a transcription factor that targets antioxidant response element and regulates the antioxidant gene expression.^42^ Dysregulation of NRF2 antioxidant defense signaling in endothelial cells may be associated with the endotheliopathy associated with COVID-19 infection.^43^ Soluble vascular cell adhesion molecule-1 (sVCAM-1) is a protein that plays a key role in fibrosis and cardiac remodeling^44^ and migration of leukocytes to the vessels,^45^ and it may act in endothelial dysfunction.^46^ Serum levels of sVCAM-1 were higher than healthy control regarding COVID-19 patients.^47^ Increased inflammation, platelet dysfunction, and imbalances in the coagulation mechanism in COVID-19 infection have caused disruptions in the normal coagulation mechanism.^48^ Besides d-dimer, fibrinogen, and different molecules are thought to be a part of this dysregulation. Endothelial damage and dysregulation of angiogenesis may lead to microthrombus formation and organ damage, and hence ARDS can be considered a marker of COVID-19 infection.^49^ Adropin has been a newly identified bioactive peptide hormone.^50^ The relationship between adropin and inflammation has been studied before^51^; however the level of adropin was first demonstrated by Aydın et al^18^ in COVID-19 patients. Adropin level was found to be lower in patients with COVID-19 compared to healthy controls. In addition, this reduction was observed even less in COVID-19 patients with diabetes mellitus than in patients with only COVID-19.^18^ Endothelial cell-specific molecule 1 (endocan) is a proteoglycan involved in inflammatory processes .^52^ Pascreau et al^53^ showed the presence of higher endocan levels in COVID-19 patients compared to the control group. They demonstrated the presence of a significant increase in endocan level from day 1 to day 3 in COVID-19 patients with mild/moderate ARDS after hospitalization. Angiopoietin-2 (Ang-2) is a member of angiopoietin/Tie (tyrosine kinase with immunoglobulin and epidermal growth factor (EGF) homology domains) signaling pathway that has a main role in angiogenesis.^54^ Ang-2/Ang-1 level measured in COVID-19 patients. Ang-2/Ang-1 ratio was higher in non-survivor COVID-19 patients than survivor COVID-19 patients.^55^ Vascular system factors are perhaps among the most important factors in this disease. They are vital not only in the disease stage but also in the post-COVID process.

### Lung-Related Molecules

The lungs are one of the primary targets of COVID-19 infection due to their ACE2 receptors and dense vascular surface. Increased amount of cytokines, fibroblast proliferation, endothelial and epithelial damage, and hyaline membrane formation cause hypoxia in the lungs.^9^ Appelberg et al^56^ performed an integrative proteo-transcriptomics analysis in Huh7 cells infected with SARS-CoV-2 virus. The authors demonstrated that ErbB, hypoxia-inducible factor-1α (HIF-1 α), mammalian target of rapamycin (mTOR), and TNF pathways were more modulated in SARS-CoV-2 infection compared to pathways. Hypoxia-inducible factor-1α is a molecule that is induced by increased reactive oxygen species production due to SARS-CoV-2 infection and enhances the inflammatory response.^57^ In our recent study, we investigated the mRNA expression of HIF-1α in COVID-19 patients. Higher HF-1α expression was determined in severe COVID-19 patients than healthy individuals.^16^ This may be a sign of decreased oxygen supply in the lungs as well as a sign of course in COVID-19 infection.

Transforming growth factor-β is a cytokine that exerts profibrogenic, anti-inflammatory, and immunosuppressive effects during and after both sepsis and COVID-19 infection.^58^ Connective tissue growth factor (CTGF) is a protein that is involved in fibroblast growth and extracellular matrix production, and it is induced by TGF-β.^59,60^ Fibronectin-1 is an extracellular matrix protein that plays a role in wound healing and oncogenic transformation.^61,62^ It has important roles in the growth, differentiation, and migration of cells.^61,62^ In the a bioinformatics study, the authors declared that SARS-CoV-2 infection leads to increasing ACE2, transforming growth factor beta 1 (TGFB1), CTGF, and fibronectin-1 mRNA that are involved in lung fibrosis.^63^ Surfactant protein D (SP-D) is a member of collectin family proteins and synthesized by type 2 alveolar epithelium. Kerget et al^10^ showed that SP-D protein level was higher in COVID-19 patients with MAS and ARDS than patients without MAS and ARDS. Kidney injury molecule-1 (KIM-1) is a molecule whose expression is increased after kidney injury, directing cells to apoptosis.^64^ Kidney injury molecule-1, which has also been shown to be present in the lung tissue, acts as a receptor mediating the entry of the SARS-CoV-2 virus into the cell.^65^ Kidney injury molecule-1 level was found to be considerably higher in COVID-19 patients compared to healthy individuals. In addition, it has been shown that it is found at a higher level in severe patients than in moderate patients.^66^ Kidney injury molecule-1 has been measured in COVID-19 patients as an indicator of kidney health. It was observed that the KIM-1 level was relatively higher in surviving patients than in deceased patients.^67^ In addition, it has been shown that it is found at a higher level in severe patients than in moderate patients.^68^ Soluble urokinase plasminogen activator receptor (suPAR) is soluble form of urokinase plasminogen activator which is released at condition of inflammation, sepsis, kidney disease, cancer, and coronary artery disease.^69,70^ Urokinase plasminogen activator has a role in angiogenesis, inflammatory response, migration, adhesion, and proliferation.^71^ In COVID-19 infection, the suPAR level was increased in moderate patients; however, in severe patients a lower suPAR level was observed when compared to moderate patients.^66^ An increased suPAR level in COVID-19 patients has also been demonstrated in another study.^72^ Furthermore, increased levels of suPAR were associated with stage 1, stage 2, and stage 3 acute kidney insuffiencey.^73^ Numerous lung-related molecular dysregulations are observed in COVID-19 infection. Considering that the lungs are one of the primary targets for SARS-CoV-2, it may be easily said that there are many more molecular changes.

### Immune Molecules

The immune system may overreact as it fights to defend the host from viral and bacterial infections and it may damage cells, tissues, and organs.^74,75^ The study by Chen et al^27^ has shown that IFN signaling is high from the onset of the COVID-19 disease process to the hospitalization period. Interleukin-6, IL-10, and IL-8 levels were higher in severe patients compared to mild patients. Upregulated genes involved in neutrophil extracellular traps contribute to disease pathogenesis.^27^ Increased T cell signaling at the onset of the disease may be lost in the stages of the disease. Several other molecules and pathways including IL-17 pathway and p38 MAPK activation were involved in COVID-19 infection.^27^ Increased metabolic activity in the immune system may lead to upregulation or downregulation in some molecule expressions.^76,77^ Interleukin-18 is a cytokine that is a member of the IL-1 family and is involved in IFN-gamma synthesis in natural killer cells and T cells.^78^ Interleukin-1 receptor antagonist (IL-1Ra) is a protein that inhibits the activity of IL-1, which has an important role in the cytokine storm.^79^ Alpha defensin is another cytokine that defends the cell against viral and bacterial infections.^11^ Kerget et al^11^ demonstrated increased IL-18, IL-1Ra, and alpha defensing levels within COVID-19 patients with MAS and COVID-19 patients with ARDS compared with the control group. Tumor necrosis factor-α is a cytokine that is localized upstream of the inflammation cascade and has multiple roles in the immune system.^80^ A higher TNF-α level was shown in severe COVID-19 patients than in healthy individuals.^81^ Progranulin (PGRN) is a growth factor involved in normal tissue development, regeneration, proliferation, and host defense.^82^ Endothelial cells and immune system cells secrete PRG in case of infections in the body.^83,84^ Due to its role in the immune system, it has been the focus of many studies including infectious diseases.^85,86^ Özgeriş et al^86^ demonstrated higher PGRN levels in COVID-19 patients when compared to healthy individuals. Their receiver operating characteristic (ROC) analysis results showed that PRGN discriminated COVID-19 patients from healthy individuals. Yao et al^47^ also revealed increased PGRN levels in COVID-19 patients when compared to healthy individuals. Macrophage migration inhibitory factor is a molecule that is expressed in many cells, and it stimulates several cytokine productions including IFN-γ, TNF-α, IL-6, and IL-1β.^87^ Macrophage migration inhibitory factor was found in high levels in severe COVID-19 patients compared with the control group.^12^ The triggering receptor expressed on myeloid cell (TREM) family expressed many cells including immune cells. The triggering receptor expressed on myeloid cells have roles in inflammation and proinflammatory cytokine discharge.^9^ The triggering receptor expressed on myeloid cell 1 and TREM2 levels were measured in COVID-19 patients, and the results revealed that COVID-19 patients had higher TREM1 and TREM2 levels than healthy controls. The triggering receptor expressed on myeloid cell 1 levels were higher in severe patients than in moderate patients.^9^ This indicates that increased TREM1 may be a sign of disease severity in COVID-19 infection. .^88,89^ The level of glucose-regulated protein gp96 (gp96), is a heat shock protein, induced by infection or inflammation leads to the activation of the immune system and the release of cytokines.^90,91^ Plasma gp96 level has been associated with IL-6 and disease severity in COVID-19 patients.^92^ Plasma gp96 had reliable power to distinguish non-serious COVID-19 patients from severe COVID-19 patients.^92^ Immune system molecules are among the most studied molecules in studies where molecular biomarkers are sought. Immune overactivation and cytokine storm are part of the COVID-19 infection. Every molecule that can play a role in these mechanisms is a candidate to be a biomarker.

## Discussion

In this review, we summarized disease-associated molecules from multiple studies of COVID-19 infection. We see that the genes studied in COVID-19 infection are mostly multifunctional.^11,93^ No molecule has a single function.^94^ Therefore, it is possible to come across many studies of these genes in more than 1 disease or mechanism.^95^ Hypoxia-inducible factor-1α molecule is a molecule whose expression is increased in hypoxia. However, hypoxia is not only seen in COVID-19 infection. Although endocan has been seen as a marker of endothelial damage in COVID-19, it can also be encountered as a part of the disease mechanism in non-infective diseases.^94^ In fact, by arranging their studies, the researchers prioritized the study of molecules that had previously been shown to be effective in viral or bacterial diseases and even cancer.^95^ Therefore, most of the molecules that have been shown to function for COVID-19 and have important roles in this infection are already functional in other diseases. This is an important data for revealing the common mechanisms of COVID-19 infection with other diseases. If these intersections are more clearly revealed, existing drugs^20^ can be used or modified instead of finding a new drug for COVID-19 infection. 

Studies have shown that there are changes in the expressions of molecules that are components of many mechanisms in COVID-19 infection.^2,28^ As there are molecules with increased expression, the number of molecules with decreased expression is also quite high.^77^ It is also conceivable that the common points here are symptoms rather than molecules. Many diseases contain similar symptoms. Hypoxia, endothelial damage, cytokine storm, respiratory failure, and excessive immune response are pathophysiology that can be seen in many diseases.^96,97^ It is also possible to move forward with this perspective when investigating disease-related molecules or treatments. The molecular mechanisms of these pathologies have been studied for years. However, the presence of a large number of molecules involved in the pathogenesis of the disease makes it difficult to identify a biomarker for the disease. Considering all the changes in the data obtained, the presence of a large number of molecules makes the diagnosis and prognosis of the disease difficult. Instead of a single biomarker, it may be more accurate to consider the mechanisms and pathways of potential biomarkers collectively. But this is both costly and difficult. Databases that can be developed for this can facilitate this work. In addition, studies were mostly carried out on patient groups. When viewed within the framework of individual medicine, a patient-specific gene expression panel may be studied to develop a patient-specific treatment, and what the patient needs and does not need can be evaluated in this way. Like gene expression panels, which are frequently used in cancer diagnosis, gene panels can be created in such infective diseases.

One of the situations we see in the COVID-19 infection is that the disease or the effects of the disease are not completely healed after treatment. Getting rid of the severe effects of the disease or being discharged from the hospital does not mean getting rid of the disease completely. The effects of the disease can last for a very long time.^98^ Elimination of the virus does not completely remove the damage caused by the disease, which leads to the emergence of post-COVID symptoms. In particular, cardiovascular system-related disorders are among the conditions that occur frequently in the post-COVID period.^99^ While doing molecular marker research, perhaps by separating the disease into periods, additional studies may be done for periodic markers.

## Conclusion

Genetic susceptibility analysis for pre-disease, disease-onset molecular changes, disease severity-related molecular changes, molecular changes for the recovery period, and molecular changes for post-COVID may be considered one by one. In order to reveal the role of a single molecule in the entire disease process and in the post-disease process, it can be measured and followed-up at regular intervals to show the disease-specific periodic change of a single biomarker. Dysregulated innate or adaptive immunity in SARS-CoV-2 infection might occasionally trigger an MAS picture, which awaits full molecular elucidation. In the last 2 years, many studies have been carried out on SARS-CoV-2 infection, and many mechanisms have been tried to be clarified. However, there are still many molecular changes that need to be investigated. Future studies will continue to update themselves with the mutations of the virus.

## References

[1] Karabulut Uzuncakmak S Dirican E Naldan ME Kesmez Can F Halıcı Z . Investigation of CYP2E1 and caspase-3 Gene expressions in COVID-19 patients. Gene Rep. 2022;26:101497. (10.1016/j.genrep.2022.101497) 35071821 PMC8760173

[2] Kerget B Kerget F Kızıltunç A et al. Evaluation of the relationship of serum vitamin D levels in COVID-19 patients with clinical course and prognosis. Tuberk Toraks. 2020;68(3):227 235. (10.5578/tt.70027) 33295720

[3] Ozden A Doneray H Hafize Erdeniz EH Altinkaynak K Igan H . Clinical and laboratory findings by serum vitamin D levels in children with COVID-19. Eurasian J Med. 2022;54(3):285 291. (10.5152/eurasianjmed.2022.22213) 36301009 PMC9797750

[4] Rauti R Shahoha M Leichtmann-Bardoogo Y et al. Effect of SARS-CoV-2 proteins on vascular permeability. eLife. 2021;10. (10.7554/eLife.69314) PMC8545399 34694226

[5] Kesmez Can F Özkurt Z Öztürk N Sezen S . Effect of IL‐6, IL‐8/CXCL8, IP‐10/CXCL 10 levels on the severity in COVID 19 infection. Int J Clin Pract. 2021;75(12):e14970. (10.1111/ijcp.14970) 34626520 PMC8646602

[6] Bikdeli B Madhavan MV Jimenez D et al. COVID-19 and thrombotic or thromboembolic disease: implications for prevention, antithrombotic therapy, and follow-up: JACC State-of-the-art review. J Am Coll Cardiol. 2020;75(23):2950 2973. (10.1016/j.jacc.2020.04.031) 32311448 PMC7164881

[7] Kerget F Kerget B Kahraman ÇY et al. Evaluation of the relationship between PENTRAXIN 3 (PTX3) rs2305619 (281A/G) and rs1840680 (1449A/G) polymorphisms and the clinical course of COVID-19. J Med Virol. 2021;93(12):6653 6659. (10.1002/jmv.27238) 34314051 PMC8426891

[8] Gonzales JN Lucas R Verin AD . The acute respiratory distress syndrome: mechanisms and perspective therapeutic approaches. Austin J Vasc Med. 2015;2(1). PMC4786180 26973981

[9] Kerget F Kerget B İba Yılmaz S Kızıltunç A . Evaluation of the relationship between TREM‐1/TREM‐2 ratio and clinical course in COVID‐19 pneumonia. Int J Clin Pract. 2021;75(10):e14697. (10.1111/ijcp.14697) 34365706 PMC8420347

[10] Kerget B Kerget F Koçak AO et al. Are serum interleukin 6 and surfactant protein D levels associated with the clinical course of COVID-19? Lung. 2020;198(5):777 784. (10.1007/s00408-020-00393-8) 32918573 PMC7486805

[11] Kerget B Kerget F Aksakal A Aşkın S Sağlam L Akgün M . Evaluation of alpha defensin, IL‐1 receptor antagonist, and IL‐18 levels in COVID‐19 patients with macrophage activation syndrome and acute respiratory distress syndrome. J Med Virol. 2021;93(4):2090 2098. (10.1002/jmv.26589) 33038012 PMC7675303

[12] Aksakal A Kerget B Kerget F Aşkın S . Evaluation of the relationship between macrophage migration inhibitory factor level and clinical course in patients with COVID‐19 pneumonia. J Med Virol. 2021;93(12):6519 6524. (10.1002/jmv.27189) 34241898 PMC8426684

[13] McGonagle D Sharif K O’Regan A Bridgewood C . The role of cytokines including interleukin-6 in COVID-19 induced pneumonia and macrophage activation syndrome-like disease. Autoimmun Rev. 2020;19(6):102537. (10.1016/j.autrev.2020.102537) 32251717 PMC7195002

[14] Du Y Tu L Zhu P et al. Clinical features of 85 fatal cases of COVID-19 from wuhan. A retrospective observational study. Am J Respir Crit Care Med. 2020;201(11):1372 1379. (10.1164/rccm.202003-0543OC) 32242738 PMC7258652

[15] Yevgi R Relationship between acute phase reactants and disability in Guillian-Barré syndrome during the COVID-19 pandemic. Arch Med Res. 2022;53(2):179 185. (10.1016/j.arcmed.2021.10.002) 34690009 PMC8520852

[16] Karabulut Uzunçakmak S Naldan ME Dirican E Kerget F Halıcı Z . Preliminary investigation of gene expression levels of PAPP-A, STC-2, and HIF-1α in SARS-Cov-2 infected patients. Mol Biol Rep. 2022;49(9):8693 8699. (10.1007/s11033-022-07710-9) 35796937 PMC9261127

[17] Ticinesi A Lauretani F Nouvenne A et al. C-reactive protein (CRP) measurement in geriatric patients hospitalized for acute infection. Eur J Intern Med. 2017;37:7 12. (10.1016/j.ejim.2016.08.026) 27594414

[18] Aydın P Uzunçakmak SK Tör İH Bilen A Özden A . Comparison of serum adropin levels in patients with diabetes mellitus, COVID-19, and COVID-19 with diabetes mellitus. Eurasian J Med. 2022;54(2):197 201. (10.5152/eurasianjmed.2022.22128) 35703530 PMC9634878

[19] Kerget B Aksakal A Kerget F . Evaluation of the relationship between laboratory parameters and pulmonary function tests in COVID‐19 patients. Int J Clin Pract. 2021;75(7):e14237. (10.1111/ijcp.14237) 33864318 PMC8250304

[20] Kerget B Kerget F Aydın M Karaşahin Ö . Effect of montelukast therapy on clinical course, pulmonary function, and mortality in patients with COVID‐19. J Med Virol. 2022;94(5):1950 1958. (10.1002/jmv.27552) 34958142 PMC9015221

[21] Schwalfenberg GK A review of the critical role of vitamin D in the functioning of the immune system and the clinical implications of vitamin D deficiency. Mol Nutr Food Res. 2011;55(1):96 108. (10.1002/mnfr.201000174) 20824663

[22] Grant WB Lahore H McDonnell SL et al. Evidence that vitamin D supplementation could reduce risk of influenza and COVID-19 infections and deaths. Nutrients. 2020;12(4):988. (10.3390/nu12040988) PMC7352449 32492787

[23] Khurana D Deoke SA . Thrombocytopenia in critically ill patients: clinical and laboratorial behavior and its correlation with short-term outcome during hospitalization. Indian J Crit Care Med. 2017;21(12):861 864. (10.4103/ijccm.IJCCM_279_17) 29307969 PMC5752797

[24] Hui P Cook DJ Lim W Fraser GA Arnold DM . The frequency and clinical significance of thrombocytopenia complicating critical illness: a systematic review. Chest. 2011;139(2):271 278. (10.1378/chest.10-2243) 21071526

[25] Tang N Bai H Chen X Gong J Li D Sun Z . Anticoagulant treatment is associated with decreased mortality in severe coronavirus disease 2019 patients with coagulopathy. J Thromb Haemost. 2020;18(5):1094 1099. (10.1111/jth.14817) 32220112 PMC9906401

[26] Xu P Zhou Q Xu J . Mechanism of thrombocytopenia in COVID-19 patients. Ann Hematol. 2020;99(6):1205 1208. (10.1007/s00277-020-04019-0) 32296910 PMC7156897

[27] Chen YM Zheng Y Yu Y et al. Blood molecular markers associated with COVID‐19 immunopathology and multi‐organ damage. EMBO J. 2020;39(24):e105896. (10.15252/embj.2020105896) 33140861 PMC7737620

[28] Karabulut Uzunçakmak S Aksakal A Kerget F Aydın P Halıcı Z . Evaluation of IGFBP5 expression and plasma osteopontin level in COVID-19 patients. Adv Med Sci. 2022;68(1):31 37. (10.1016/j.advms.2022.11.001) 36427358 PMC9640409

[29] Hoffmann M Kleine-Weber H Schroeder S et al. SARS-CoV-2 cell entry depends on ACE2 and TMPRSS2 and is blocked by a clinically proven protease inhibitor. Cell. 2020;181(2):271 280.e8. (10.1016/j.cell.2020.02.052) 32142651 PMC7102627

[30] Abdelsattar S Kasemy ZA Ewida SF et al. ACE2 and TMPRSS2 SNPs as determinants of susceptibility to, and severity of, a COVID-19 infection. Br J Biomed Sci. 2022;79:10238. (10.3389/bjbs.2021.10238) 35996506 PMC8915702

[31] Möhlendick B Schönfelder K Breuckmann K et al. ACE2 polymorphism and susceptibility for SARS-CoV-2 infection and severity of COVID-19. Pharmacogenet Genomics. 2021;31(8):165 171. (10.1097/FPC.0000000000000436) 34001841 PMC8415730

[32] Ponti G Pastorino L Manfredini M et al. COVID‐19 spreading across world correlates with C677T allele of the methylenetetrahydrofolate reductase (MTHFR) gene prevalence. J Clin Lab Anal. 2021;35(7):e23798. (10.1002/jcla.23798) 34061414 PMC8209953

[33] Hamming OJ Terczyńska-Dyla E Vieyres G et al. Interferon lambda 4 signals via the IFNλ receptor to regulate antiviral activity against HCV and coronaviruses. EMBO J. 2013;32(23):3055 3065. (10.1038/emboj.2013.232) 24169568 PMC3844954

[34] Saponi-Cortes JMR Rivas MD Calle-Alonso F et al. IFNL4 genetic variant can predispose to COVID-19. Sci Rep. 2021;11(1):21185. (10.1038/s41598-021-00747-z) PMC8551167 34707167

[35] Zhang C Wu Z Li JW Zhao H Wang GQ . Cytokine release syndrome in severe COVID-19: interleukin-6 receptor antagonist tocilizumab may be the key to reduce mortality. Int J Antimicrob Agents. 2020;55(5):105954. (10.1016/j.ijantimicag.2020.105954) 32234467 PMC7118634

[36] Kerget F Kerget B . Frequency of interleukin-6 rs1800795 (−174G/C) and rs1800797 (−597G/A) polymorphisms in COVID-19 patients in turkey who develop macrophage activation syndrome. Jpn J Infect Dis. 2021;74(6):543 548. (10.7883/yoken.JJID.2021.046) 33952771

[37] Gu SX Tyagi T Jain K et al. Thrombocytopathy and endotheliopathy: crucial contributors to COVID-19 thromboinflammation. Nat Rev Cardiol. 2021;18(3):194 209. (10.1038/s41569-020-00469-1) 33214651 PMC7675396

[38] Ackermann M Verleden SE Kuehnel M et al. Pulmonary vascular endothelialitis, thrombosis, and angiogenesis in Covid-19. N Engl J Med. 2020;383(2):120 128. (10.1056/NEJMoa2015432) 32437596 PMC7412750

[39] O’Sullivan JM Gonagle DM Ward SE Preston RJS O’Donnell JS . Endothelial cells orchestrate COVID-19 coagulopathy. Lancet Haematol. 2020;7(8):e553 e555. (10.1016/S2352-3026(20)30215-5) 32619412 PMC7326442

[40] Pine AB Meizlish ML Goshua G et al. Circulating markers of angiogenesis and endotheliopathy in COVID‐19. Pulm Circ. 2020;10(4):2045894020966547. (10.1177/2045894020966547) 33282193 PMC7691906

[41] Goshua G Pine AB Meizlish ML et al. Endotheliopathy in COVID-19-associated coagulopathy: evidence from a single-centre, cross-sectional study. Lancet Haematol. 2020;7(8):e575 e582. (10.1016/S2352-3026(20)30216-7) 32619411 PMC7326446

[42] Kansanen E Kivelä AM Levonen AL . Regulation of Nrf2-dependent gene expression by 15-deoxy-Δ12,14-prostaglandin J2. Free Radic Biol Med. 2009;47(9):1310 1317. (10.1016/j.freeradbiomed.2009.06.030) 19573595

[43] Ungvari Z Tarantini S Nyúl-Tóth Á et al. Nrf2 dysfunction and impaired cellular resilience to oxidative stressors in the aged vasculature: from increased cellular senescence to the pathogenesis of age-related vascular diseases. GeroScience. 2019;41(6):727 738. (10.1007/s11357-019-00107-w) 31655958 PMC6925097

[44] Schnabel RB Larson MG Yamamoto JF et al. Relations of biomarkers of distinct pathophysiological pathways and atrial fibrillation incidence in the community. Circulation. 2010;121(2):200 207. (10.1161/CIRCULATIONAHA.109.882241) 20048208 PMC3224826

[45] Ziliotto N Zivadinov R Jakimovski D et al. Relationships among circulating levels of hemostasis inhibitors, chemokines, adhesion molecules, and MRI characteristics in multiple sclerosis. Front Neurol. 2020;11:553616. (10.3389/fneur.2020.553616) 33178104 PMC7593335

[46] Contreras-Briceño F Herrera S Vega-Adauy J et al. Circulating vascular cell adhesion Molecule-1 (sVCAM-1) is associated with left atrial remodeling in long-distance runners. Front Cardiovasc Med. 2021;8:737285. (10.3389/fcvm.2021.737285) 34790706 PMC8591189

[47] Yao S Luo N Liu J et al. Elevated serum levels of progranulin and soluble vascular cell adhesion molecule-1 in patients with COVID-19. J Inflamm Res. 2021;14:4785 4794. (10.2147/JIR.S330356) 34584437 PMC8464378

[48] Robba C Battaglini D Ball L et al. Coagulative disorders in critically ill COVID-19 patients with acute distress respiratory syndrome: a critical review. J Clin Med. 2021;10(1):140. (10.3390/jcm10010140) PMC7795033 33401632

[49] Pons S Fodil S Azoulay E Zafrani L . The vascular endothelium: the cornerstone of organ dysfunction in severe SARS-CoV-2 infection. Crit Care. 2020;24(1):353. (10.1186/s13054-020-03062-7) PMC7296907 32546188

[50] Kumar KG Trevaskis JL Lam DD et al. Identification of adropin as a secreted factor linking dietary macronutrient intake with energy homeostasis and lipid metabolism. Cell Metab. 2008;8(6):468 481. (10.1016/j.cmet.2008.10.011) 19041763 PMC2746325

[51] Bozic J Borovac JA Galic T Kurir TT Supe-Domic D Dogas Z . Adropin and inflammation biomarker levels in male patients with obstructive sleep apnea: a link with glucose metabolism and sleep parameters. J Clin Sleep Med. 2018;14(7):1109 1118. (10.5664/jcsm.7204) 29991422 PMC6040799

[52] Béchard D Scherpereel A Hammad H et al. Human endothelial-cell specific Molecule-1 binds directly to the integrin CD11a/CD18 (LFA-1) and blocks binding to intercellular adhesion molecule-1. J Immunol. 2001;167(6):3099 3106. (10.4049/jimmunol.167.6.3099) 11544294

[53] Pascreau T Tcherakian C Zuber B Farfour E Vasse M Lassalle P . A high blood endocan profile during COVID-19 distinguishes moderate from severe acute respiratory distress syndrome. Crit Care. 2021;25(1):166. (10.1186/s13054-021-03589-3) PMC8101335 33957951

[54] Akwii RG Sajib MS Zahra FT Mikelis CM . Role of angiopoietin-2 in vascular physiology and pathophysiology. Cells. 2019;8(5):471. (10.3390/cells8050471) PMC6562915 31108880

[55] Villa E Critelli R Lasagni S et al. Dynamic angiopoietin-2 assessment predicts survival and chronic course in hospitalized patients with COVID-19. Blood Adv. 2021;5(3):662 673. (10.1182/bloodadvances.2020003736) 33560382 PMC7876870

[56] Appelberg S Gupta S Svensson Akusjärvi S et al. Dysregulation in Akt/mTOR/HIF-1 signaling identified by proteo-transcriptomics of SARS-CoV-2 infected cells. Emerg Microbes Infect. 2020;9(1):1748 1760. (10.1080/22221751.2020.1799723) 32691695 PMC7473213

[57] Tian M Liu W Li X et al. HIF-1α promotes SARS-CoV-2 infection and aggravates inflammatory responses to COVID-19. Signal Transduct Target Ther. 2021;6(1):308. (10.1038/s41392-021-00726-w) PMC8371950 34408131

[58] Russell B Moss C George G et al. Associations between immune-suppressive and stimulating drugs and novel COVID-19—a systematic review of current evidence. Ecancermedicalscience. 2020;14:1022. (10.3332/ecancer.2020.1022) 32256705 PMC7105343

[59] Blom IE Goldschmeding R Leask A . Gene regulation of connective tissue growth factor: new targets for antifibrotic therapy? Matrix Biol. 2002;21(6):473 482. (10.1016/s0945-053x(02)00055-0) 12392758

[60] Moussad EE-DA Brigstock DR . Connective tissue growth factor: what’s in a name? Mol Genet Metab. 2000;71(1-2):276 292. (10.1006/mgme.2000.3059) 11001822

[61] Pankov R Yamada KM . Fibronectin at a glance. J Cell Sci. 2002;115(20):3861 3863. (10.1242/jcs.00059) 12244123

[62] Ritzenthaler JD Han S Roman J . Stimulation of lung carcinoma cell growth by fibronectin–integrin signalling. Mol Biosyst. 2008;4(12):1160-1169. (10.1039/b800533h) 19396378

[63] Xu J Xu X Jiang L Dua K Hansbro PM Liu G . SARS-CoV-2 induces transcriptional signatures in human lung epithelial cells that promote lung fibrosis. Respir Res. 2020;21(1):182. (10.1186/s12931-020-01445-6) PMC7359430 32664949

[64] Bonventre JV Kidney injury molecule-1: a translational journey. Trans Am Clin Climatol Assoc. 2014;125:293 299. 25125746 PMC4112686

[65] Yutaro Mori CF et al. KIM-1/TIM-1 is a receptor for SARS-CoV-2 in lung and kidney. medRxiv Prepr. Serv. Heal Sci. 2022.

[66] Kerget B Kerget F Aksakal A Aşkın S Uçar EY Sağlam L . Evaluation of the relationship between KIM‐1 and suPAR levels and clinical severity in COVID‐19 patients: a different perspective on suPAR. J Med Virol. 2021;93(9):5568 5573. (10.1002/jmv.27099) 34019703 PMC8242801

[67] Bezerra GF Meneses GC Albuquerque PL et al. Urinary tubular biomarkers as predictors of death in critically ill patients with COVID-19. Biomark Med. 2022;16(9):681 692. (10.2217/bmm-2021-0631) 35531623 PMC9083946

[68] Vogel MJ Mustroph J Staudner ST et al. Kidney injury molecule-1: potential biomarker of acute kidney injury and disease severity in patients with COVID-19. J Nephrol. 2021;34(4):1007 1018. (10.1007/s40620-021-01079-x) 34110585 PMC8190170

[69] Aronen A Aittoniemi J Huttunen R et al. Plasma suPAR may help to distinguish between chronic pancreatitis and pancreatic cancer. Scand J Gastroenterol. 2021;56(1):81 85. (10.1080/00365521.2020.1849383) 33245246

[70] Hodges G Lyngbæk S Selmer C et al. SuPAR is associated with death and adverse cardiovascular outcomes in patients with suspected coronary artery disease. Scand Cardiovasc J. 2020;54(6):339 345. (10.1080/14017431.2020.1762917) 32400206

[71] Smith HW Marshall CJ . Regulation of cell signalling by uPAR. Nat Rev Mol Cell Biol. 2010;11(1):23 36. (10.1038/nrm2821) 20027185

[72] Lippi G Henry BM Favaloro EJ . Elevated soluble urokinase plasminogen activator receptor (suPAR) in COVID-19 patients. Clin Chem Lab Med. 2021;59(11):e413 e415. (10.1515/cclm-2021-0561) 34116589

[73] Azam TU Shadid HR Blakely P et al. Soluble urokinase receptor (SuPAR) in COVID-19–related AKI. J Am Soc Nephrol. 2020;31(11):2725 2735. (10.1681/ASN.2020060829) 32963090 PMC7608953

[74] Newton AH Cardani A Braciale TJ . The host immune response in respiratory virus infection: balancing virus clearance and immunopathology. Semin Immunopathol. 2016;38(4):471 482. (10.1007/s00281-016-0558-0) 26965109 PMC4896975

[75] Cicchese JM Evans S Hult C et al. Dynamic balance of pro‐ and anti‐inflammatory signals controls disease and limits pathology. Immunol Rev. 2018;285(1):147 167. (10.1111/imr.12671) 30129209 PMC6292442

[76] Sharif-Askari FS Sharif-Askari NS Hafezi S et al. Interleukin-17, a salivary biomarker for COVID-19 severity. PLoS One. 2022;17(9):e0274841. (10.1371/journal.pone.0274841) PMC9498944 36136963

[77] Atila A Alay H Yaman ME et al. The serum amino acid profile in COVID-19. Amino Acids. 2021;53(10):1569 1588. (10.1007/s00726-021-03081-w) 34605988 PMC8487804

[78] Dinarello CA Novick D Kim S Kaplanski G . Interleukin-18 and IL-18 binding protein. Front Immunol. 2013;4:289. (10.3389/fimmu.2013.00289) 24115947 PMC3792554

[79] Vaninov N In the eye of the COVID-19 cytokine storm. Nat Rev Immunol. 2020;20(5):277 277. (10.1038/s41577-020-0305-6) PMC7132547 32249847

[80] Wu F Zhao S Yu B et al. A new coronavirus associated with human respiratory disease in China. Nature. 2020;579(7798):265 269. (10.1038/s41586-020-2008-3) 32015508 PMC7094943

[81] Li M Guo W Dong Y et al. Elevated exhaustion levels of NK and CD8+ T cells as indicators for progression and prognosis of COVID-19 disease. Front Immunol. 2020;11:580237. (10.3389/fimmu.2020.580237) 33154753 PMC7591707

[82] Sun R Wang D Song Y Li Q Su P Pang Y . Granulin as an important immune molecule involved in lamprey tissue repair and regeneration by promoting cell proliferation and migration. Cell Mol Biol Lett. 2022;27(1):64. (10.1186/s11658-022-00360-6) PMC9338584 35907821

[83] Zou S Luo Q Song Z et al. Contribution of progranulin to protective lung immunity during bacterial pneumonia. J Infect Dis. 2017;215(11):1764 1773. (10.1093/infdis/jix197) 28595330

[84] Luo Q Yan X Tu H Yin Y Cao J . Progranulin aggravates pulmonary immunopathology during influenza virus infection. Thorax. 2019;74(3):305 308. (10.1136/thoraxjnl-2018-211916) 30228207

[85] Jian J Konopka J Liu C . Insights into the role of progranulin in immunity, infection, and inflammation. J Leukoc Biol. 2013;93(2):199 208. (10.1189/jlb.0812429) 23089745 PMC3545674

[86] Özgeriş FB Koçak ÖF Kurt N Parlak E Yüce N Keles MS . High serum progranulin levels in COVID-19 patients: a pilot study. Biochemistry (Mosc). 2022;87(3):207 214. (10.1134/S0006297922030026) 35526852 PMC8916789

[87] Nobre CCG de Araújo JM Fernandes TA et al. Macrophage migration inhibitory factor (MIF): biological activities and relation with cancer. Pathol Oncol Res. 2017;23(2):235 244. (10.1007/s12253-016-0138-6) 27771887

[88] Tsan MF Gao B . Heat shock proteins and immune system. J Leukoc Biol. 2009;85(6):905 910. (10.1189/jlb.0109005) 19276179

[89] Land WG Role of DAMPs in respiratory virus-induced acute respiratory distress syndrome—with a preliminary reference to SARS-CoV-2 pneumonia. Genes Immun. 2021;22(3):141 160. (10.1038/s41435-021-00140-w) 34140652 PMC8210526

[90] Liu W Chen M Li X et al. Interaction of toll-like receptors with the molecular chaperone Gp96 is essential for its activation of cytotoxic T lymphocyte response. PLoS One. 2016;11(5):e0155202. (10.1371/journal.pone.0155202) PMC4868323 27183126

[91] Yang Y Li Z . Roles of heat shock protein gp96 in the ER quality control: redundant or unique function? Mol Cells. 2005;20(2):173 182. 16267390

[92] Wei R Zhou B Li S et al. Plasma gp96 is a novel predictive biomarker for severe COVID-19. Microbiol Spectr. 2021;9(3):e0059721. (10.1128/Spectrum.00597-21) 34817280 PMC8612155

[93] Janbandhu V Tallapragada V Patrick R et al. Hif-1a suppresses ROS-induced proliferation of cardiac fibroblasts following myocardial infarction. Cell Stem Cell. 2022;29(2):281 297.e12. (10.1016/j.stem.2021.10.009) 34762860 PMC9021927

[94] Lv Y Zhang Y Shi W et al. The association between endocan levels and subclinical atherosclerosis in patients with type 2 diabetes mellitus. Am J Med Sci. 2017;353(5):433 438. (10.1016/j.amjms.2017.02.004) 28502328

[95] Mu G Zhu Y Dong Z Shi L Deng Y Li H . Calmodulin 2 facilitates angiogenesis and metastasis of gastric cancer via STAT3/HIF-1A/VEGF-A mediated macrophage polarization. Front Oncol. 2021;11:727306. (10.3389/fonc.2021.727306) 34604066 PMC8479158

[96] Tamburello A Bruno G Marando M . COVID-19 and pulmonary embolism: not a coincidence. Eur J Case Rep Intern Med. 2020;7(6):001692. (10.12890/2020_001692) 32523920 PMC7279901

[97] Todros T Paulesu L Cardaropoli S et al. Role of the macrophage migration inhibitory factor in the pathophysiology of pre-eclampsia. Int J Mol Sci. 2021;22(4):1823. (10.3390/ijms22041823) PMC7917653 33673075

[98] Lechner-Scott J Levy M Hawkes C Yeh A Giovannoni G . Long COVID or post COVID-19 syndrome. Mult Scler Relat Disord. 2021;55:103268. (10.1016/j.msard.2021.103268) 34601388 PMC8447548

[99] Silva Andrade B Siqueira S de Assis Soares WR et al. Long-COVID and post-COVID health complications: an up-to-date review on clinical conditions and their possible molecular mechanisms. Viruses. 2021;13(4):700. (10.3390/v13040700) PMC8072585 33919537

